# Effect of the ethnic, profession, gender, and social background on the perception of upper dental midline deviations in smile esthetics by Chinese and Black raters

**DOI:** 10.1186/s12903-023-02893-4

**Published:** 2023-04-14

**Authors:** Mazen Musa, Riham Awad, Abdalla Mohammed, Hibatalrahman Abdallah, Mohamed Elhoumed, Leena Al-waraf, Wanting Qu, Najah Alhashimi, Xi Chen, Shuang Wang

**Affiliations:** 1 Key Laboratory of Shaanxi Province for Craniofacial Precision Medicine Research, College of Stomatology, Xi’an Jiaotong University, Xi’an, People’s Republic of China; 2 Laboratory Center of Stomatology, College of Stomatology, Xi’an Jiaotong University, Xi’an, People’s Republic of China; 3 Department of Orthodontics, College of Stomatology, Xi’an Jiaotong University, Xi’an, People’s Republic of China; 4 Department of Stomatology, The First Affiliated Hospital of Xi’an Jiaotong University, Xi’an, Shaanxi 710004 People’s Republic of China; 5 Department of Orthodontics, Al Tagana Dental Teaching Hospital, Faculty of Dentistry, University of Science and Technology Omdurman, Omdurman, Khartoum 11111 Sudan; 6 Department of Pediatrics dentistry, College of Stomatology, Xi’an Jiaotong University, Xi’an, Shaanxi People’s Republic of China; 7 University of Science and Technology Omdurman, Khartoum, Sudan; 8 Department of Prosthodontics, University of Khartoum, Khartoum, Sudan; 9 Department of Epidemiology and Biostatistics, School of Public Health, Xi’an Jiaotong University, Xi’an, People’s Republic of China; 10 National Institute of Public Health Research (INRSP), BP. 695, Nouakchott, Mauritania; 11 Unit and Divisional Chief Orthodontics at Hamad Medical Corporation, and Associate Professor, College of Dental Medicine, Qatar University, Doha, Qatar

**Keywords:** Smile, Layperson, Likert scale, Midline coincidence, Orthodontist

## Abstract

**Background:**

The purpose of this study was to compare the perception of upper dental midline deviation on the attractiveness of a smile among raters from different ethnicities, professions, genders, and ages and measure to what extent the presence or absence of the associated smiling structures influence the raters' evaluations.

**Methods:**

A male subject (26 years of age) with adequate smile characteristics was selected by 3 experienced orthodontists, and 561 raters from 2 different ethnic groups (281 Chinese raters and 280 Black raters) rated the subject's smile after the subject's upper dental midline was digitally altered from 0 to 5 mm using a 5-point Likert scale on 12 smile photographs divided into two groups: group 1, in the presence of smile related structures, two-thirds of the nose, lips, and chin (NLC), and group 2, in the absence of smile related structures, the lips only (L).

**Results:**

There were statistically significant differences (*p* < 0.05) between the two ethnicities, in 2 mm and 4 mm in-group NLC and 5 mm in-group L, as well as the raters' profession to each midline shift of both groups (NLC) and (L) for both ethnicities except for 0 mm. Regarding the role of associated smile structures, the smile photos were observed in the presence of smile-associated structures, and in its absence (NLC × L), statistically significant differences (*p* < 0.05) were found when the deviation was 5 mm among the Chinese raters; in 1 mm, and 4 mm among the Black raters. Among different genders, statistical differences were only reported (*p* < 0.05) for Chinese raters for 5 mm in NLC, while statistical differences were observed for 2 mm and 3 mm in NLC for Black raters. For age categories, differences were observed (*p* < 0.05) for 4 mm, 5 mm in NLC and 4 mm, and 5 mm in L for Chinese raters, while 5 mm in NLC and 1 mm in L for Black raters.

**Conclusion:**

Perception of the upper dental midline deviations was influenced by the factors of ethnicity, profession, presence or absence of smile-associated structures, as well as the gender and age of the raters.

**Supplementary Information:**

The online version contains supplementary material available at 10.1186/s12903-023-02893-4.

## Background

Dento-facial aesthetics play a fundamental part in modern dental practice, as seen by patients' rising requests for more cosmetic and aesthetic procedures [1]. However, beauty perceptions vary according to desires and are influenced by ethnocultural background [2]. A smile is a common facial expression that expresses pleasantness and friendship [2]. According to neurological control, there are two types of smiles: involuntary (spontaneous), associated with emotion, and voluntary (posed), which is not usually associated with emotion [3]. Throughout interpersonal interactions, people depend and focus primarily on the mouth and the eyes of the other person [4]. While examining facial aesthetics, coordination of the teeth is more critical than the eyes [5], and smile-associated structures like the chin play a role in facial beauty, where its prominence suggests "strength" while its reduction suggests "weakness" in a man's personality [6].

Furthermore, it is believed that the nose, because of its prominent position in the face, significantly affects how an observer perceives the face [7]. People with ideal smiles are perceived to be more knowledgeable and are more likely to have a career than those without ideal smiles [8]. Additionally, there is evidence that people's self-confidence and self-esteem might suffer when they have an unattractive face [6], where an ideal smile is seen as an important factor in attractiveness.

The analysis of a smile involves an appreciation of the patient's harmony between facial and dental midlines, the existence of buccal corridor space (BCS), smile arc, the amount of gingival exposure, teeth proportionality, teeth color, occlusal plane inclination, and aesthetics of gingiva [9]. The threshold at which normal asymmetry becomes abnormal is a matter of clinical judgment and patient perception [10]. Adjusting the dental-to-facial midline disparity is tough: it may add to the complexity and time of orthodontic therapy [11]. Moreover, orthodontists' aesthetic judgment does not always match and correspond to the patient's perspective [10].

Different ethnic groups perceive smiles differently. For instance, the dental protrusion is a common and acceptable feature in Afro-Caribbean patients. However, the same feature could be viewed as unattractive by Caucasians. Differences also exist with reference to gender. Compared to men, women typically prefer more upper gingival exposure when smiling. Males, however, are less picky than females when judging a smile; this implies that there are gender differences in terms of tolerance levels and emphasizes the value of taking the individual patient's concerns into account when performing smile analysis [2]. Additionally, since different cultures may have different norms for these smile features, ethnicity must be considered a critical variable.

Multiple investigations have attempted to determine the acceptable limit of upper dental midline deviations. That said, the results are still controversial [12–19]. Photographs [20], videos [21, 22], three-dimensional (3D) stereo-photogrammetric pictures [23], and using eye-tracking programs [24] are some methods for assessing a smile. Furthermore, numerous studies employed full-face photos to measure the upper dental midline deviations [12, 13, 25]. Some researchers examined laypeople's deviations in a photo showing a smile only [14–16], and by contrast, [10] another researcher utilized two sets of photos: one with half of the face and one with just the smile.

To the best of our knowledge, both Chinese and Black ethnic groups have no recorded findings on the perception of the upper dental midline comparing dental and non-dental professionals in the presence or absence of smile-associated structures. Thus, an element of this study is involved with contributing to gaps in the literature.

### Objectives

The objective of the present study was to compare the perception of upper dental midline deviation (in relation to the facial midline) on the attractiveness of a smile among raters from different ethnicities (Chinese and Black) and professions (laypeople, art students, senior dental students, general dental practitioners, and orthodontists), genders, and social backgrounds (ages) and measure to what extent the presence or absence of associated smiling structures (nose and chin) influence the raters’ evaluations on a digitally altered smile.

## Material and methods

### Study design

This cross-sectional study was approved by The Institutional Review Board Committee at the Hospital of stomatology of Xi'an Jiaotong University (xjkgll [2019] n0.015), and informed consent was obtained from all participants. In addition, the University of Science and Technology in Sudan approved the study.

This study was carried out in China at Xi'an Jiaotong University and in Sudan at the University of Science and Technology Omdurman. The authors used a simple random sample procedure; raters were also recruited from other universities' yards, hostels, hospitals, private clinics, and offices from August 2019 to December 2019.

### Sample size calculations

The sample size was calculated using G*Power (V. 3.1.9.4) based on a previous study that reported a mean attractiveness score of (2.22 ± 0.94) and (2.18 ± 0.81) by laypeople and orthodontists respectively, assuming a small effect size difference (0.25) between groups. The power analysis showed a total sample size estimate of 220 raters required for each ethnic group, with at least 45 participants for each group based on the profession at a conventional α level (0.05) and desired power (1 – β) of 0.85 [26]. This number was later increased to 280 for each ethnic group.

### Selection criteria

The inclusion criteria of raters were: 1) Adults 18 and above; 2) Only Chinese individuals who lived in China or Sudanese individuals who lived in Sudan; 3) Individuals with no dental education or orthodontic treatment for non-dental professionals (laypeople and art students); 4) University graduates for laypeople; 5) Dental and art students in their final year; 6) General practitioners and orthodontists with at least 2 years of experience.

### Smile

**Fig. 1:**
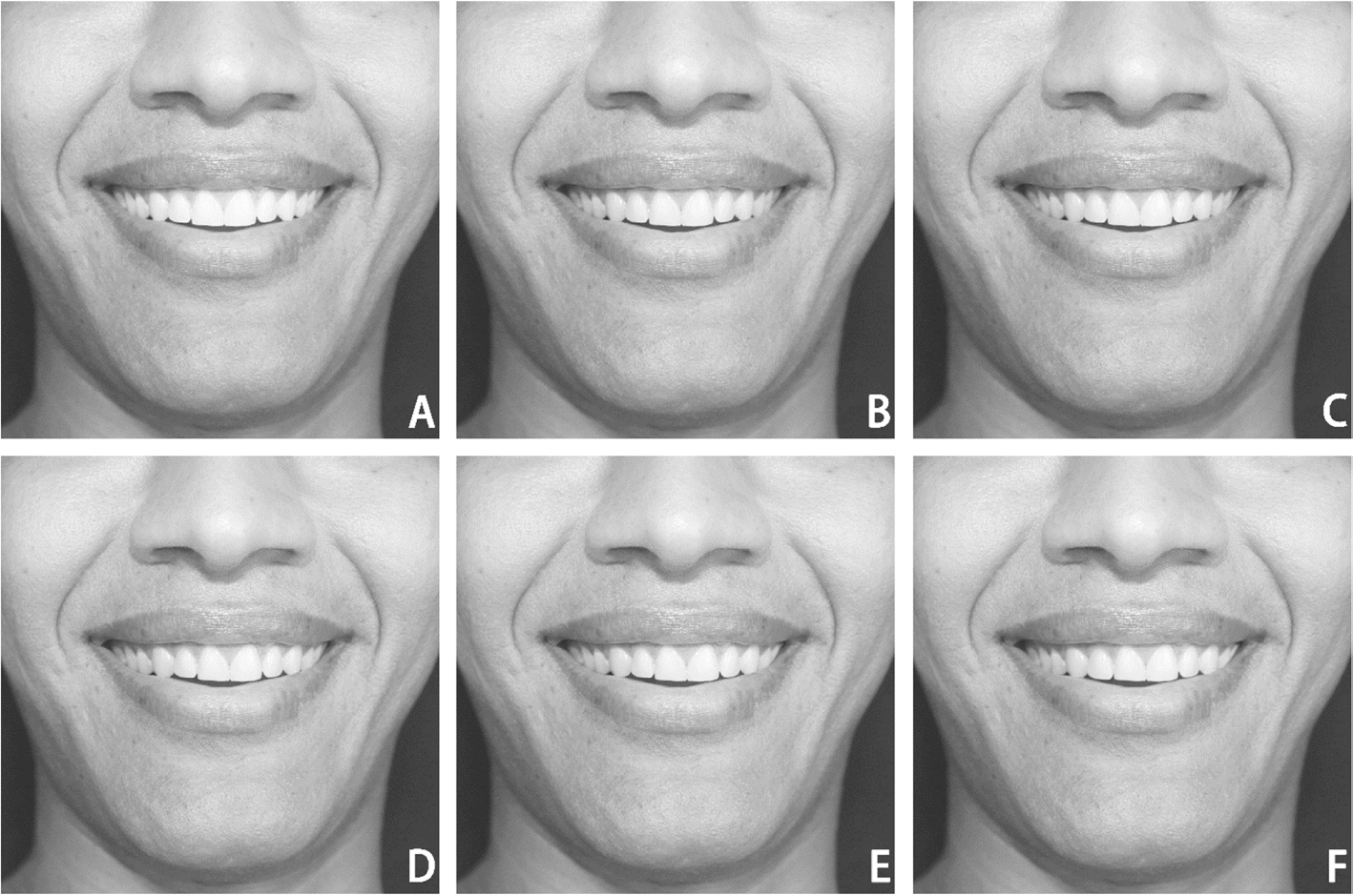
Group (NLC) photographs: show the Maxillary dental midline was altered to the left side as follows (**A)** ideal; (**B)** 1-mm; (**C)** 2-mm; (**D)** 3-mm; (**E)** 4-mm; (**F)** 5-mm

**Fig. 2:**
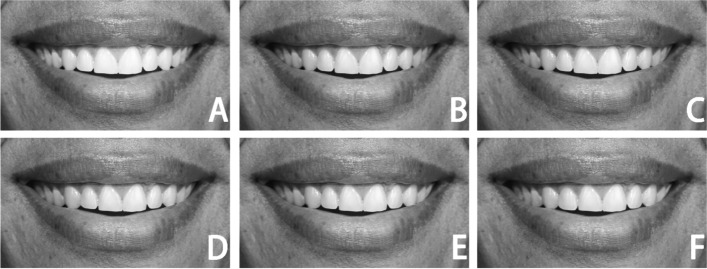
Group (L) photographs: show the Maxillary dental midline was altered to the left side as follows (**A)** ideal; (**B)** 1-mm; (**C)) **2-mm; (**D)** 3-mm; (**E)** 4-mm; (**F)** 5-mm

Photograph Preparations:

- Step 1: Find an appropriate subject among three candidates. A male subject (26 years of age) with adequate smile characteristics was selected by three experienced orthodontists.
- Step 2: Capture a photo. The subject was photographed smiling indoors by a professional photographer with adequate light and black studio backdrops and two studio strobes, using a digital camera (EOS 1300D, Canon; Tokyo, Japan) fixed on a tripod and a standardized focus on a frontal pose of the subject in a sitting position. The subject was 5 feet away from the camera lens, with the subject's head in a natural posture.
- Step 3: Select an appropriate photo. Any photos displaying head rotations along the vertical axis with imprecise clinical marks without reasonable resolution were excluded. The same experienced orthodontists selected one photo among the best three photos.
- Step 4: Adjust the photos. The chosen photo was then subsequently imported to Adobe Photoshop (CS5.1, San Jose, Calif.) and was cropped and divided into two groups: group 1, which included two-thirds of the nose, lips, and chin (NLC), and group 2, which only included the smiling lips (L) [10]. Afterward, the photos were converted to black and white [26].
- Step 5: Alter the midline. Constant incremental changes in the upper dental midline were generated with the advancement of 1 mm each time, from 0 to 5 mm to the left side, to the facial midline (The center of the top lip or philtrum, employed as a representative of the facial midline).
- Step 6: Print the photo. Photos were designed and curated to replicate the subject's original scale and size (real size scale) [10]. The researcher assigns a code to each photograph, which is then printed on glossy paper and organized randomly in an album in the following order: 3, 2, 0, 5, 4, and 1 mm (Fig. 1) and (Fig. 2).

### Questionnaire

This study employed a printed questionnaire on A4 paper, included a brief overview of the study as well as information about the gender, age, education, and profession of the raters followed by three questions: "How vital an attractive smile for raters, rater's satisfaction with their smile, and the smile's impact on society's acceptance." A five-point Likert scale was employed (1 = very high, 2 = high, 3 = medium, 4 = low, and 5 = very low) for scoring [9].

Before evaluating the photos, the raters were shown two calibration photos, one with a 0 mm (ideal) deviation and the other with a 6 mm deviation to the left side. However, the results of those photos were not taken into account [10].

Each page contained a single smiling photo, followed by 12 photographs to judge (0 to 5 mm); raters rated as follow: (1 = very attractive, 2 = attractive, 3 = accepted, 4 = unattractive, and 5 = very unattractive).

In an office with adequate light, the raters were interviewed individually and viewed the photographs at a gap distance of roughly 30 cm. Raters were reminded not to contrast photos while assessing smiles, and each photograph was viewed for 20 s, with a 10-s interval between photos to allow raters to score on the questionnaire [10].

### Statistical analysis

Statistical analysis was performed using SPSS 25.0 software (IBM, Chicago Inc., US). The questionnaire and photo reliability were evaluated using intra-class correlation coefficients (ICC) by a random subset of 200 raters from both ethnicities who were asked two weeks later to reassess the same questionnaire and photos. After the data were checked for normality, a Mann–Whitney test was used to investigate the influence of the associated structures of the smile on the perception of upper dental midline deviations and compare different ethnicities, age groups, and gender. The Kruskal Wallis test was also used in order to assess the effect of alterations in the upper dental midline on the esthetic perception of the smile in addition to comparing the professions of raters in NLC and L, followed by the Wilcoxon test to compare paired groups. Pearson correlation coefficient and regression equations were developed in order to determine the association between deviations in NLC and L and the mean values of raters. For each group, a descriptive statistic of the mean and standard deviation (SD) is provided, with the result's significance set at (*p* < 0.05).

## Results

### Study demographic

This study had a total of 561 raters, consisting of 281 Chinese raters and 280 Black raters. By age and gender, the study consisted of 282 females and 279 males, where 425 raters were in the 18–30 age range, and 136 raters were in the 31–51 years age range, with 26 years and 6 months as the average age. In terms of profession, 112 orthodontists, 112 general practitioners, 112 dental students, 113 art students, and 112 laypersons participated in the study [More details of the study demographic are described in Table 1].

**Table 1:** Show the demographic data of the raters

	**Overall raters**		**Chinese raters**		**Black raters**	
**Profession**	**n**	**mean age**	**n**	**mean age**	**n**	**mean age**
Orthodontists	112	35.9 ± 5.5	56	36.8 ± 5.5	56	35 ± 5.5
G.Practitioners	112	26.4 ± 5.2	56	25 ± 5.9	56	27.8 ± 3.8
Dental students	112	22.1 ± 1.4	56	21.8 ± 1.1	56	22.4 ± 1.6
Art students	113	22.6 ± 4	57	20.4 ± 3.3	56	24.9 ± 3.3
Laypersons	112	26.1 ± 6	56	23.1 ± 4	56	20.2 ± 6.2
Total	561 (100%)	-	281 (50.1%)	-	280 (49.9%)	-
**Gender**	**n**	**%**	**n**	**%**	**n**	**%**
Male	279	(49.7%)	123	(43.8%)	156	(55.7%)
Female	282	(50.3%)	158	(56.2%)	124	(44.3%)
Total	561	(100%)	281	(100%)	280	(100%)
**Age group**	**n**	**%**	**n**	**%**	**n**	**%**
18–30	425	(75.8%)	219	(77.9%)	206	(73.6%)
31–51	136	(24.2%)	62	(22.1%)	74	(26.4%)
Total	561	(100%)	281	(100%)	280	(100%)

Furthermore, the earlier and later scores for the questionnaire and photos showed an intraclass correlation coefficient ranging from 0.83 to 0.90 [see Additional File 1].

### Smile

The mean value of all raters for an attractive smile (ideal) was (2.3 ± 0.41), which was identified as the cut-off point score for what was considered attractive; smiles that raters scored less than or equal to the above-mentioned mean value were considered attractive, while smiles that raters scored as higher than that were deemed as less attractive.

### Questionnaire

For the three questions related to the smile regarding the appreciation of an appealing smile, Chinese orthodontists and Black art students perceived a more attractive smile as more crucial than other groups. For question two regarding satisfaction with the smile, Chinese laypeople and Black art students were most satisfied with their smiles compared to other raters. Concerning question three and the effect of the smile on social acceptability, Chinese senior dental students and Black art students rated the influence of smile attractiveness on social acceptance as more significant in comparison to other groups. Overall, there were significant differences between all groups for the three questions (*p* < 0.001) for both ethnic groups [see Additional File 2].

### Overall population and ethnic groups

There was statistical significance (*p* < 0.05) for gender across both ethnic groups in 2 and 3 mm in NLC as well as in age groups in 1 mm NLC and 4 and 5 mm in both NLC and L. In addition, significance was observed between the two ethnic groups in 2 and 4 mm in NLC and 5 mm in L. Regarding deviation detection ability, Chinese raters identified deviation at approximately 3 mm in both NLC and L, while Black raters detected deviation starting from 2 mm in both NLC and L (Table 2; [see Additional File 3. A, B, C, D, E, F]).

**Table 2:** Attractiveness scores mean and significance of gender, age, and ethnicity by overall raters (*n* = 561)

**Deviation**		**NLC scores for overall raters**						**L scores for overall raters**						***P*-value**	
(mm)	Male (*n* = 279) mean ± SD	Female (*n* = 282) mean ± SD	18-30 years (*n* = 112) mean ± SD	31-51 years (*n* = 112) mean ± SD	Chinese (*n* = 281) mean ± SD	Black (*n* = 280) mean ± SD	Male (*n* = 279) mean ± SD	Female (*n* = 282) mean ± SD	18-30 years (*n* = 425) mean ± SD	31-51 years (*n* = 136) mean ± SD	Chinese (*n* = 281) mean ± SD	Black (*n* = 280) mean ± SD	Across gender	Across age groups	Across ethnicity
0	2.2 ± 0.9	2.2 ± 0.8	2.3 ± 0.9	2.2 ± 0.9	2.2 ± 0.8	2.3 ± 1.0	2.1 ± 0.8	2.2 ± 0.8	2.2 ± 0.8	2.2 ± 0.8	2.2 ± 0.8	2.2 ± 0.9	n.s	n.s	n.s
1	2.3 ± 0.9	2.2 ± 0.9	2.2 ± 0.9	2.5 ± 0.9	2.3 ± 0.9	2.3 ± 0.9	2.1 ± 1.0	2.3 ± 1.1	2.2 ± 1.0	2.3 ± 1.1	2.3 ± 1.0	2.1 ± 0.9	n.s	* NLC	n.s
2	2.3 ± 0.9	2.2 ± 0.9	2.3 ± 0.9	2.4 ± 0.9	2.2 ± 0.9	2.4 ± 0.8	2.2 ± 0.9	2.3 ± 0.8	2.2 ± 0.9	2.4 ± 1.1	2.3 ± 0.4	2.4 ± 1.0	* NLC	n.s	* NLC
3	2.5 ± 0.9	2.4 ± 0.9	2.5 ± 0.9	2.6 ± 0.9	2.5 ± 0.9	2.5 ± 0.8	2.3 ± 1.0	2.4 ± 1.0	2.4 ± 1.0	2.6 ± 1.1	2.4 ± 1.0	2.5 ± 1.0	* NLC	* L	n.s
4	2.5 ± 0.9	2.7 ± 0.9	2.6 ± 0.9	3.0 ± 1.0	2.6 ± 0.9	2.8 ± 1.0	2.6 ± 1.0	2.7 ± 1.2	2.6 ± 1.1	2.9 ± 1.1	2.7 ± 1.1	2.7 ± 1.1	n.s	*NLC + L	* NLC
5	2.6 ± 0.9	2.8 ± 0.8	2.7 ± 1.0	3.1 ± 0.9	2.7 ± 0.9	2.9 ± 1.1	2.6 ± 1.0	2.7 ± 0.9	2.7 ± 1.0	3.0 ± 1.1	2.6 ± 1.0	2.9 ± 1.0	n.s	*NLC + L	* L

A higher score implies a less attractive smile

^*^Mean it is significant (*P* < 0.05); *n.s* not significant in both NLC and L

### Effect of profession

Statistically significant (*p* < 0.05) differences were observed for each digitally shifted midline of both groups NLC and L across the different professions for both ethnic groups except for the 0 mm. Orthodontists detected deviations starting from 1 mm in NLC and L for both ethnic groups. In contrast, Chinese general practitioners detected deviations in 3, 4, and 5 mm in NLC and 4 and 5 mm in L, compared to 4 and 5 mm in NLC and 3 and 5 mm in L for Black raters.

Additionally, Chinese senior dental students detected deviations at 3 mm for both NLC and L, while Black raters rated all photos as unattractive. Chinese art students detected deviations starting from 3 mm in both NLC and L, except they rated 0 mm in NLC as unattractive, while Black Art students detected deviations of 3 and 4 mm in NLC and 2 and 5 mm in L only.

In terms of laypeople, Chinese laypeople did not detect the deviation at all in both NLC and L, while Black Laypeople detected it only at 4 mm in NLC and 5 mm in L (Table 3; [see Additional File 4, 5 (A, B)]).

**Table 3:** Attractiveness scores mean and significance of profession and role of associated structures (NLC, L) by Chinese raters (*n* = 281) and Black raters (*n* = 280)

Deviation	NLC scores for Chinese raters					L scores for Chinese raters					*p*-value	
(mm)	Orthodontists (*n* = 56) mean ± SD	G. Practitioners (*n* = 56) mean ± SD	Dental students (*n* = 56) mean ± SD	Art students (*n* = 57) mean ± SD	Layperson (*n* = 56) mean ± SD	Orthodontists (*n* = 56) mean ± SD	G.Practitioners (*n* = 56) mean ± SD	Dental students (*n* = 56) mean ± SD	Art students (*n* = 57) mean ± SD	Layperson (*n* = 56) mean ± SD	Across profession	NLC x L Results*
0	2.2 ± 0.8	2.2 ± 0.8	2.2 ± 0.9	2.4 ± 0.8	2.0 ± 0.9	2.2 ± 0.8	2.2 ± 0.8	2.1 ± 0.9	2.3 ± 0.9	2.2 ± 0.8	n.s	n.s
1	2.6 ± 0.9	2.5 ± 1.0	2.0 ± 0.7	2.3 ± 0.9	2.0 ± 0.9	2.6 ± 0.9	2.2 ± 1.0	2.0 ± 0.7	2.3 ± 0.9	2.2 ± 0.9	* NLC + L	n.s
2	2.6 ± 0.9	2.3 ± 0.7	1.9 ± 0.8	2.1 ± 0.4	2.1 ± 0.9	2.6 ± 1.0	2.3 ± 0.8	2.0 ± 0.8	2.0 ± 0.9	2.3 ± 0.8	* NLC + L	n.s
3	2.6 ± 1.1	2.5 ± 0.9	2.5 ± 0.7	2.6 ± 1.0	2.2 ± 0.9	2.9 ± 1.1	2.1 ± 1.0	2.4 ± 0.8	2.4 ± 1.2	1.9 ± 0.7	* NLC + L	n.s
4	3.3 ± 0.8	2.7 ± 0.9	2.5 ± 0.8	2.5 ± 1.0	2.2 ± 0.9	3.2 ± 0.9	2.5 ± 0.9	2.7 ± 0.9	2.8 ± 1.6	2.3 ± 0.8	* NLC + L	n.s
5	3.1 ± 0.9	2.9 ± 0.8	2.7 ± 0.8	2.6 ± 0.9	2.3 ± 0.8	3.2 ± 0.8	2.5 ± 1.0	2.7 ± 0.9	2.5 ± 0.9	2.3 ± 1.0	* NLC + L	*
**Deviation**	**NLC scores for Black raters**					**L scores for Black raters**					***p*-value**	
(mm)	Orthodontists (*n* = 56) mean ± SD	G. Practitioners (*n* = 56) mean ± SD	Dental students (*n* = 56) mean ± SD	Art students (*n* = 56) mean ± SD	Laypersons (*n* = 56) mean ± SD	Orthodontists (*n* = 56) mean ± SD	G. Practitioners (*n* = 56) mean ± SD	Dental students (*n* = 56) mean ± SD	Art students (*n* = 56) mean ± SD	Laypersons (*n* = 56) mean ± SD	Across profession	NLC x L Results*
0	2.3 ± 0.9	2.3 ± 0.8	2.5 ± 1.5	2.2 ± 0.8	2.2 ± 0.8	2.2 ± 0.8	1.9 ± 0.9	2.4 ± 0.8	2.1 ± 0.8	2.2 ± 0.9	n.s	*
1	2.6 ± 0.7	2.1 ± 1.0	2.5 ± 0.8	2.1 ± 0.9	2.3 ± 0.8	2.7 ± 1.0	1.5 ± 0.7	2.5 ± 0.8	2.2 ± 0.9	2.0 ± 0.8	* NLC + L	*
2	2.7 ± 0.8	2.3 ± 0.8	2.6 ± 1.2	2.3 ± 0.6	2.2 ± 1.1	2.6 ± 1.0	2.0 ± 0.9	2.5 ± 1.1	2.6 ± 0.9	2.1 ± 1.0	* NLC + L	n.s
3	2.8 ± 0.7	2.3 ± 0.8	3.0 ± 0.8	2.4 ± 0.8	2.2 ± 0.8	2.8 ± 0.8	2.4 ± 0.8	2.9 ± 0.9	2.1 ± 1.4	2.3 ± 1.0	* NLC + L	n.s
4	3.2 ± 0.8	2.9 ± 0.9	3.1 ± 0.9	2.5 ± 0.9	2.4 ± 1.1	3.4 ± 0.9	2.2 ± 0.9	3.5 ± 0.9	2.0 ± 0.8	2.2 ± 1.0	* NLC + L	*
5	3.4 ± 0.8	2.8 ± 0.9	3.5 ± 1.4	2.3 ± 0.8	2.3 ± 1.0	3.4 ± 0.8	2.5 ± 0.9	3.8 ± 0.8	2.4 ± 0.8	2.5 ± 0.8	* NLC + L	n.s

A higher score implies a less attractive smile

^*^Mean it is significant (*P* < 0.05); *n.s* not significant in both NLC and L

### Associated smile structures

Regarding the role of associated structures, photographs from the NLC group and the L group were compared (NLC x L), and statistically significant differences (*p* < 0.05) were found among the Chinese raters when the deviation was 5 mm and when it was 0, 1, and 4 mm among the Black raters (Table 3; [see Additional File 4, 5 (**C**)]).

### Gender

There was a significant difference (*p* < 0.05) between males and females in a 5 mm midline shift in NLC among Chinese raters and a 2 and 3 mm midline shift for Black raters.

Furthermore, Chinese male and female raters detected deviation at the same level–3 mm in NLC–which existed with regards to L. in L, Chinese females detected it at 3 mm while Chinese males detected deviation at 4 mm. Meanwhile, Black males were more critical in detecting deviations in 2 mm NLC than 3 mm for females, although both Black males and female raters detected deviation at the same amount in 3 mm in L (Table 4; [see Additional File 4, 5 (D, E)]).

**Table 4:** Attractiveness scores mean and significance of gender and age by Chinese raters (*n* = 281) and Black raters (*n* = 280)

Deviation	NLC scores for Chinese raters				L scores for Chinese raters				*p*-value	
(mm)	Male (*n* = 158) mean ± SD	Female (*n* = 123) mean ± SD	18–30 year (*n* = 219) mean ± SD	31–51 year (*n* = 62) mean ± SD	Male (*n* = 158) mean ± SD	Female (*n* = 123) mean ± SD	18–30 year (*n* = 219) mean ± SD	31–51 year (*n* = 62) mean ± SD	Across gender	Across age
0	2.2 ± 0.9	2.2 ± 0.8	2.3 ± 0.8	2.0 ± 0.8	2.1 ± 0.8	2.2 ± 0.8	2.2 ± 0.8	2.1 ± 0.9	n.s	n.s
1	2.3 ± 0.9	2.2 ± 0.9	2.2 ± 0.9	2.5 ± 1.0	2.1 ± 1.0	2.3 ± 1.1	2.2 ± 1.1	2.3 ± 0.9	n.s	n.s
2	2.3 ± 0.9	2.2 ± 0.9	2.2 ± 0.8	2.4 ± 1.0	2.2 ± 0.9	2.3 ± 0.8	2.2 ± 0.8	2.4 ± 1.0	n.s	n.s
3	2.5 ± 0.9	2.4 ± 0.9	2.4 ± 0.9	2.6 ± 1.1	2.3 ± 1.0	2.4 ± 1.0	2.3 ± 0.9	2.6 ± 1.2	n.s	n.s
4	2.5 ± 0.9	2.7 ± 0.9	2.5 ± 0.9	3.1 ± 1.0	2.6 ± 1.0	2.7 ± 1.2	2.6 ± 1.1	3.0 ± 1.1	n.s	*NLC + L
5	2.6 ± 0.9	2.8 ± 0.8	2.6 ± 0.8	3.0 ± 1.0	2.6 ± 1.0	2.7 ± 0.9	2.5 ± 0.9	3.0 ± 1.0	* NLC	*NLC + L
**Deviation**	**NLC scores for Black raters**				**L scores for Black raters**				***p*-value**	
(mm)	Male (*n* = 158) mean ± SD	Female (*n* = 123) mean ± SD	18–30 year (*n* = 206) mean ± SD	31–51 year (*n* = 74) mean ± SD	Male (*n* = 158) mean ± SD	Female (*n* = 123) mean ± SD	18–30 year (*n* = 206) mean ± SD	31–51 year (*n* = 74) mean ± SD	Across gender	Across age
0	2.3 ± 1.1	2.3 ± 0.8	2.3 ± 1.0	2.4 ± 0.9	2.2 ± 0.9	2.1 ± 1.0	2.1 ± 0.9	2.3 ± 0.8	n.s	n.s
1	2.3 ± 0.8	2.3 ± 0.8	2.2 ± 0.9	2.5 ± 0.8	2.2 ± 0.9	2.1 ± 1.0	2.1 ± 0.9	2.4 ± 1.0	n.s	* L
2	2.6 ± 1.0	2.3 ± 0.9	2.4 ± 1.0	2.4 ± 0.9	2.4 ± 0.9	2.4 ± 1.1	2.3 ± 1.0	2.4 ± 0.9	* NLC	n.s
3	2.7 ± 0.8	2.4 ± 0.9	2.5 ± 0.8	2.6 ± 0.8	2.5 ± 0.8	2.5 ± 1.3	2.5 ± 1.1	2.6 ± 0.8	* NLC	n.s
4	2.8 ± 0.6	2.8 ± 1.1	2.8 ± 1.0	3.0 ± 0.9	2.9 ± 1.1	2.6 ± 1.1	2.6 ± 1.1	2.8 ± 1.0	n.s	n.s
5	2.9 ± 1.1	2.8 ± 1.1	2.8 ± 1.2	3.1 ± 0.9	2.9 ± 0.9	3.0 ± 1.1	2.9 ± 1.0	3.0 ± 1.0	n.s	* NLC

A higher score implies a less attractive smile

^*^Mean it’s significant (*P* < 0.05); *n.s* not significant in both NLC and L

### Age (Social Background)

Statistical significance (*p* < 0.05) was observed between age groups in 4 and 5 mm for both NLC and L for Chinese raters. Furthermore, significance was also observed with 1 mm in L and 5 mm in NLC for Black raters.

The elder Chinese age group detected deviation at 1 mm in NLC compared to 3 mm for the younger Chinese age group, and the same thing was observed in L with 2 mm for the elder and 4 mm for the younger age group.

A similar outcome was identified with Black age groups, where the elder Black age group detected deviation at 1 mm in NLC, including 0 mm compared with 2 mm for the younger Black age group, and the same pattern was observed in L with 1 mm for the elder and 3 mm for the younger age group (Table 4; [see Additional File 4, 5 (F, G)]).

### Association between deviations in NLC and L and the raters' mean values

A Pearson product-moment correlation was conducted to examine the relationship between the deviations in (NLC and L) and raters' mean values, which indicated a Positive correlation (*r* = 0.573) among deviations in groups NLC and L, where the level of significance value was (*p* < 0.001). The mean values assigned by the raters (Pearson correlation coefficient) (*r* = 0.573), (*n* = 561). The value of the coefficient of determination (R2 = 0.29.6) and the linear regression equation (y = 0.963 + 0.597 x) was derived from the data collected for this study (*p* < 0.001) [see Additional File 6].

## Discussion

A detailed evaluation of the smile features is essential in making a treatment plan, and particularly, upper anterior teeth have high aesthetic expectations from patients [3]. Given this, the objective of the present study was to compare the perception of upper dental midline deviation on the attractiveness of a smile among raters from different ethnicities, professions, genders, and ages and measure to what extent the presence or absence of associated smiling structures influence the rater's decisions.

The current study used a posed smile since it is easily repeatable. The authors involved dental professionals since they are healthcare providers with different levels of dental education (orthodontists, general practitioners, dental students). Laypeople are the primary beneficiaries of dental treatment; this study did not include patients because their doctors may affect their perception [27]. Art students were included and specifically separated from laypeople since special attention is given to faces in art [19].

In this study, the smile photographs were converted to black and white in order to avoid skin color influencing the raters' opinions [9]. Each page of the album contained a single smiling photo to eliminate confounders, and the printed photos replicated the subject's original size to ensure all raters rate the images with exact, accurate dimensions. The photos were coded to avoid the identification of deviation rhythm, and the raters rated each photo separately and randomly. The researchers controlled the rating time, ensuring no comparison of photos and a fixed view distance for the purpose of reducing bias. Unlike Alomari et al. [26], a study regarding smiles was done based on online google form questionnaires, making it difficult to control dimensions since the raters can zoom. While [28, 29] gathered the raters in a classroom, the raters' location may have influenced their responses.

Our photos were only shifted to the left side concerning the deviation. While [12, 19] moved the upper dental midline to the right and left sides and found no significant relationship between the direction of deviations and scoring photos. In contrast, [28] stated that the midline deviation to the left was more noticeable than on the right. Furthermore, [30, 31] concluded that the dental midline's natural deviation toward the left side of the face might be more common.

For this study, the Likert scale was utilized and preferred due to its simplicity [27]. Aşik et al. [24] concluded that the eye-tracking data, Likert scale, and visual analog scale (VAS) were comparable. VAS can imply different objects to raters, where part of the scale might be used, and the rest may be neglected [9].

The result of the questionnaire suggests that attractiveness might affect social interaction in different professions; this was in line with [9, 32], which strongly emphasized the role that an attractive smile plays in promoting social acceptance.

The heterogeneity of the raters and cultural differences related to smile characteristics can explain the significance of ethnicity. Furthermore, Black Sudanese raters were more critical for the overall population than Chinese raters. Our results are comparable to Brazilian [10], Nigerian [33], Iranian [19], U.S. and Canadian [15], Moroccan [34], Saudi [35], and Korean [27].

The results demonstrate the complexity of the topic in the groups based on profession, except for orthodontists, who were more critical in detecting deviations starting from 1 mm in NLC and L. The value of 1 mm for orthodontists in our results agrees with Sadrhaghighi H. et al. [19] and Pinho et al. [36]. While Adekoya et al. mentioned that orthodontists perceived a 0.5 mm deviation [33].

General practitioners were less critical than orthodontists in detecting deviations with inconsistency between NLC and L, suggesting that an associated smile structure influenced their decision in deviations other than 5 mm, where the detecting deviation at 3 mm in L agrees with the work of Sadrhaghighi H. et al. [19] on Iranian general practitioners. Moroccan and Saudi general practitioners severely underrated all incremental deviations of the maxillary midline[34, 35]. At the same time, Nigerian general practitioners could perceive a significant difference in midline deviation when it was 1.5 mm [33].

Chinese senior dental students were more critical (3 mm for both NLC and L) than Black dental students, who rated all photos as unattractive, demonstrating a lack of knowledge comparatively. While [29] reported that two-thirds of dental students thought the asymmetry of 2 mm was normal, Alhammadi et al. [17] reported that dental students thought the asymmetry of 4.21 ± 1.13 mm was normal. At the same time, it was considered acceptable, according to [37, 38]. In contrast, 91.0% of students did not find the midline shift attractive [39].

In the current study, Chinese art students were more critical. Chinese art students detected deviations starting from 3 mm in comparison to Black Art students, who exhibited a more confused, varied rating score. Our results show that art students paid attention to large midline deviations, while [19] reported 1 mm for Iranian art students and mentioned that they did not pay attention to the upper dental midline.

Black laypeople were more critical since they detected a deviation of 4 mm compared to Chinese laypeople. These results are comparable to those described in studies [18, 36], suggesting that laypeople may not distinguish a deviation up to 4 mm. On the contrary, Ferreira et al. [10] found that Brazilian laypeople could detect upper dental midline deviations of 1 mm and above in the presence of adjacent smile structures and 2 mm and above. In addition, An et al. [27] found that Korean laypeople who got treated orthodontically perceived midline at 3 mm, while those not subject to orthodontic treatment did not detect deviation.

Springer et al. [13] proposed a maximum acceptable deviation of 3.2 mm. In contrast, Kerr et al. [14] mentioned that 2.9 mm is acceptable. In the study of McLeod et al. [15], raters decided to accept deviations up to 2.9 mm for U.S. laypeople and 1.83 mm for Canadians. However, a 2.2 mm was concluded to be acceptable in a systematic review [16]. Aşik et al. [24] suggested that a 2 mm deviation was aesthetically unpleasing. Some authors used different facial types, Tanbakuchi et al. [40] found that the maximum acceptable midline deviation in long-face and short-face patients are 2.13 ± 0.85 mm and 2.32 ± 0.83 mm, respectively. Zhang et al. [12] used different face types (square, oval, and tapered), which found that laypeople may identify deviations from 2 mm.

The presence and absence of smile-associated structures affected the perception of midline deviation and were significant in 0, 1, and 4 mm. In this regard, orthodontists were not affected by the presence or absence of smile-associated structures like the other raters. These results are comparable to those described in [10, 41], verifying the impact of structures adjacent to the smile on the perception of upper dental midline deviations in 1 mm. On the other hand, [38, 39] studied midline inclinations in an asymmetrical nose and chin and found them to influence perception. Silva and colleagues [42] observed that laypeople preferred canting of the dental midline in the same direction as the deviation of the nose and chin rather than the opposite.

For gender, Chinese females were more critical than Chinese males in L, while Black males were more critical in detecting deviations in NLC than Black females. In this regard, gender is one aspect that may impact the creation of aesthetic beauty standards [9, 24], which is also supported by Aldhorae et al. [29], who also found that males were more sensitive to the midline than females. Williams et al. [25] altered smiling photos of male and female individuals, with 2.80 ± 1.27 mm accepted for the male subject and 3.04 ± 0.90 mm for the female subject. In contrast, [20, 24, 43–46] found that the upper dental midline did not differ according to gender.

Regarding the age variable, In both ethnic groups, the elder group was more critical regarding deviation, while the younger group exhibited a greater degree of acceptance. However, this does not necessarily mean that age is a significant factor; one possible explanation for this result is that orthodontists represent most of the elder group. At the same time, according to [43, 47], age affects how attractive a smile is perceived; in comparison, [18, 46] reported no association.

Additionally, correlation indicates that the higher the deviations, the higher the score assigned by the raters, and vice-versa, which is in line with [10, 41] [see Additional File 6].

This difference between our results and previous work [12–19] may have arisen from the effect of different ethnicity, methods, and scales applied. In addition, the increment of deviations used, facial characteristics such as hair and skin color [9, 10, 33, 40], and facial type [12, 25, 40] may influence the attention levels on the perception of smile aesthetics. Therefore, this study did not include full-face photos.

Furthermore, this study focused on a single aspect of smile aesthetics (midline), with no other smile components strengthening this study, on the contrasts [13, 14, 18, 25, 48], which included more than one aspect, potentially leading to suspicious findings and producing eye fatigue. Other studies, such as [27], shifted both maxillary and mandibular midline in the same photo.

This study is essential in clinical practice since it demonstrates that excessive concern may influence orthodontists' actions, leading to unnecessary intervention. Therefore, when undergoing orthodontic treatment, orthodontists must take care not to impose appearance standards on patients. Additionally, the degree of upper dental midline variance and patient interpretation must be considered in the treatment plan to succeed in treatment in a satisfactory and sufficient manner.

This study's limitations include solely using a male smile as the only photo as the subject's gender has exhibited an effect on smile attractiveness in two ethnic groups; hence the result can not be generalized. However, this was accomplished to prevent the increased number of photos rated, which may cause a lack of interest. In addition, the economic status of laypeople was not considered.

Further studies are required to establish whether the rater's economic status affects the perception of upper dental midline deviations. Furthermore, future dentofacial esthetic evaluations should include male and female smile photographs. It would have been interesting to add a male smile with a mustache.

## Conclusions

• Perception of the upper dental midline deviations was influenced by the ethnicity, profession, presence or absence of smile-associated structures, as well as the gender and age of the raters.
• This data can aid in analyzing the smiles of people of Chinese and Black origin. Nevertheless, in order to get sufficient outcomes, each case must be evaluated on an individual basis while taking the patient's expectations and preferences into account.

## Supplementary Information

**Additional file 1.** 

**Additional file 2.** 

**Additional file 3.** 

**Additional file 4.** 

**Additional file 5.** 

**Additional file 6.**

## Data Availability

The datasets used and analyzed during the current study are available from the corresponding author upon reasonable request.

## Abbreviations

ICC: Intraclass correlation coefficient
VAS: Visual analog scale
NLC: Nose, lips, and chin
L: Lips
SPSS: Statistical Package for Social Sciences
