# Supplementary material for: Effect of the ethnic, profession, gender, and social background on the perception of upper dental midline deviations in smile esthetics by Chinese and Black raters
Source: BMC Oral Health. 2023 Apr 14;23:214. doi: 10.1186/s12903-023-02893-4 (PMC10105468; doi:10.1186/s12903-023-02893-4)
Supplement: Supplementary file 1 — Additional file 1. [file 12903_2023_2893_MOESM1_ESM.docx]

**Additional file 1.** The intra-class correlation coefficient (ICC) results for test re-test of the raters.

| Questionnaire | Value |
| --- | --- |
| Q1 | 0.85 |
| Q2 | 0.84 |
| Q3 | 0.83 |
| NLC | Value |
| 0 (mm) | 0.90 |
| 1 (mm) | 0.85 |
| 2 (mm) | 0.86 |
| 3 (mm) | 0.90 |
| 4 (mm) | 0.91 |
| 5 (mm) | 0.89 |
| L | Value |
| 0 (mm) | 0.90 |
| 1 (mm) | 0.87 |
| 2 (mm) | 0.88 |
| 3 (mm) | 0.89 |
| 4 (mm) | 0.91 |
| 5 (mm) | 0.90 |
