# Supplementary material for: Effect of the ethnic, profession, gender, and social background on the perception of upper dental midline deviations in smile esthetics by Chinese and Black raters
Source: BMC Oral Health. 2023 Apr 14;23:214. doi: 10.1186/s12903-023-02893-4 (PMC10105468; doi:10.1186/s12903-023-02893-4)
Supplement: Supplementary file 2 — Additional file 2. [file 12903_2023_2893_MOESM2_ESM.docx]

**Additional file 2.** The questionnaire’s mean and standard deviations for Chinese (n=281) and Black raters (n=280).

|  | |  | **Chinese raters** | |  |  |
| --- | --- | --- | --- | --- | --- | --- |
|  | | Orthodontists, | General practitioners | Dental students | Art students | Laypersons |
| Question |  | mean ± SD | mean ± SD | mean ± SD | mean ± SD | mean ± SD |
|  | | (n = 56) | (n = 56) | (n = 56) | (n = 57) | (n = 56) |
| How important is an attractive smile to you | | 1.41 ± 0.49 | 1.66 ± 0.72 | 1.48 ± 0.63 | 2.18 ±1.02 | 2.18 ± 0.85 |
| Are you satisfied with your smile | | 2.29 ± 0.96 | 2.38 ± 0.98 | 2.64 ± 1.08 | 2.56 ±1.00 | 2.27 ± 0.84 |
| The impact of an attractive smile on social acceptance | | 1.66 ± 0.61 | 1.63 ± 0.67 | 1.52 ± 0.60 | 2.02 ±0.91 | 1.95 ± 0.81 |
|  | |  | **Black raters** | |  |  |
|  | | Orthodontists | General practitioners | Dental students | Art students | Laypeople |
| Questions |  | mean ± SD | mean ± SD | mean ± SD | mean ± SD | mean ± SD |
|  | | (n = 56) | (n = 56) | (n = 56) | (n = 56) | (n = 56) |
| How important is an attractive smile to you | | 1.59 ± 0.78 | 2.00 ± 0.89 | 1.61 ± 0.67 | 1.57 ± 0.71 | 1.70 ± 0.71 |
| Are you satisfied with your smile | | 2.46 ± 0.97 | 2.84 ± 0.94 | 2.89 ± 0.94 | 2.05 ± 0.92 | 2.68 ± 0.74 |
| The impact of an attractive smile on social acceptance | | 1.79 ± 0.68 | 2.27 ± 0.98 | 2.05 ± 0.84 | 1.77 ± 0.95 | 2.04 ± 1.04 |

A higher score implies less impact
