# Supplementary material for: Effect of the ethnic, profession, gender, and social background on the perception of upper dental midline deviations in smile esthetics by Chinese and Black raters
Source: BMC Oral Health. 2023 Apr 14;23:214. doi: 10.1186/s12903-023-02893-4 (PMC10105468; doi:10.1186/s12903-023-02893-4)
Supplement: Supplementary file 3 — Additional file 3. [file 12903_2023_2893_MOESM3_ESM.docx]

| 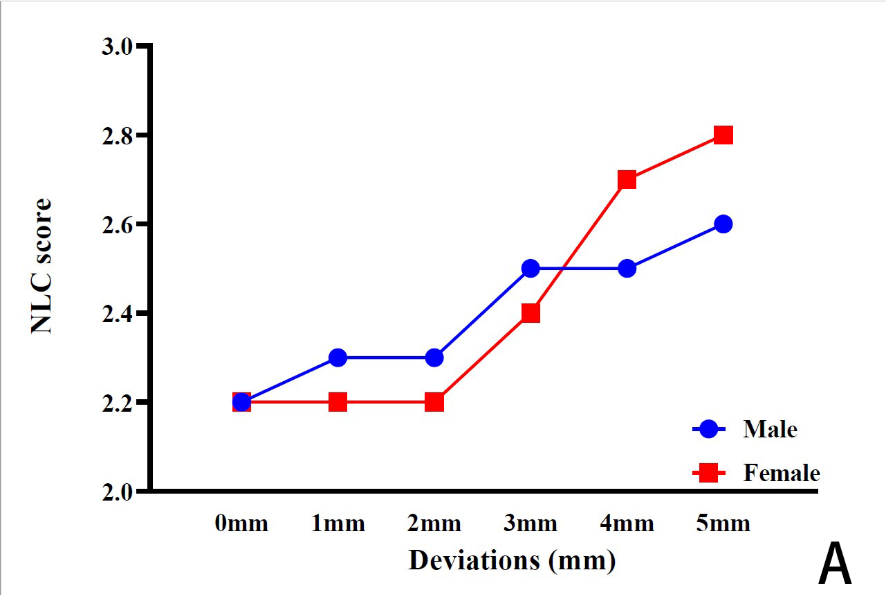 | 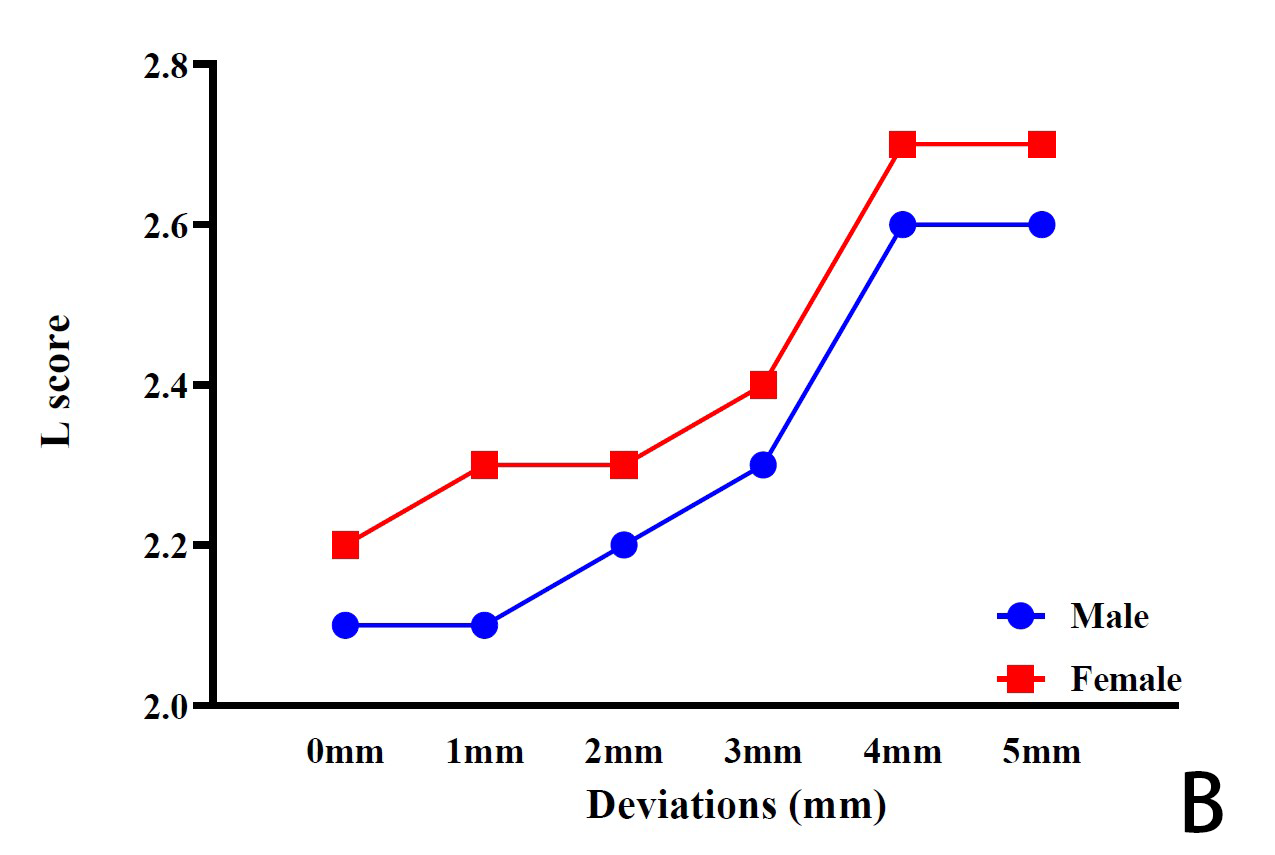 |
| --- | --- |
| 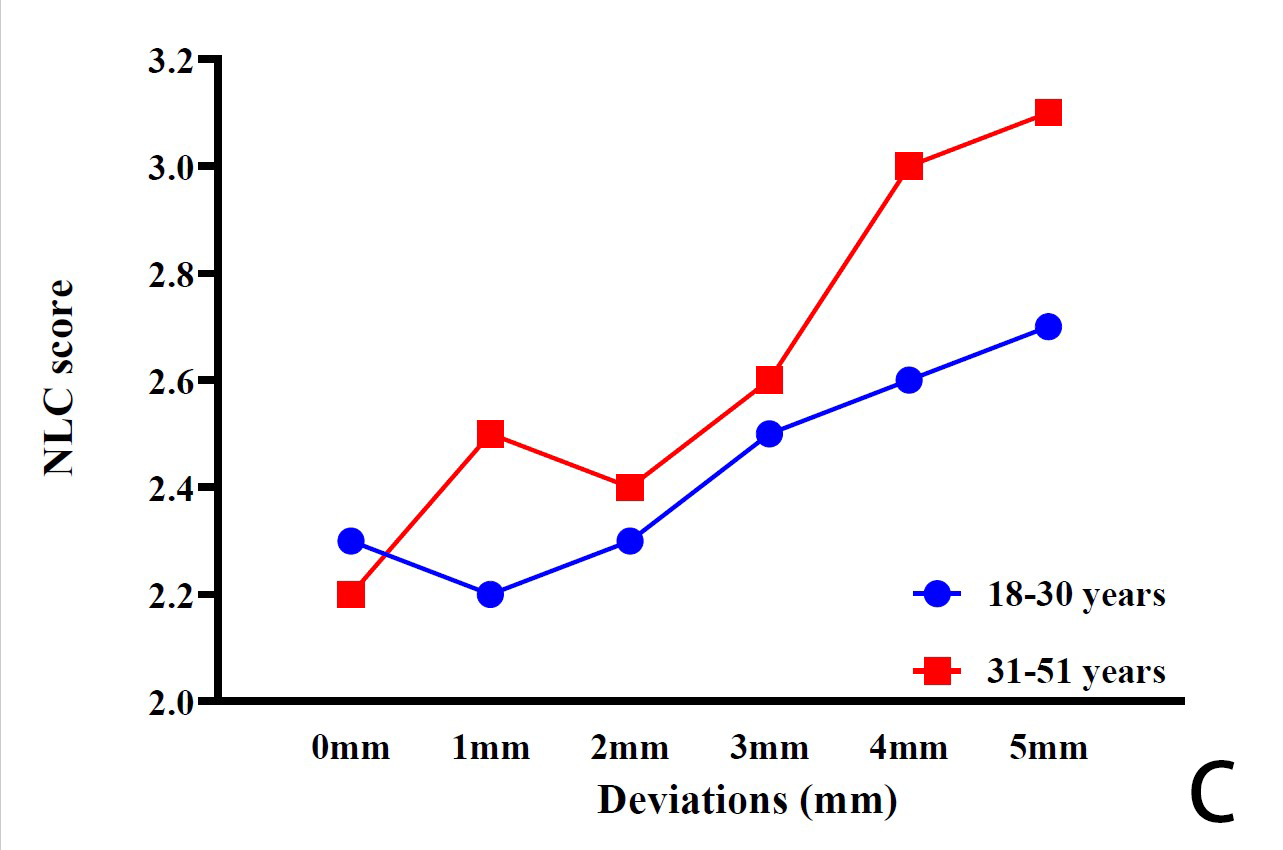 | 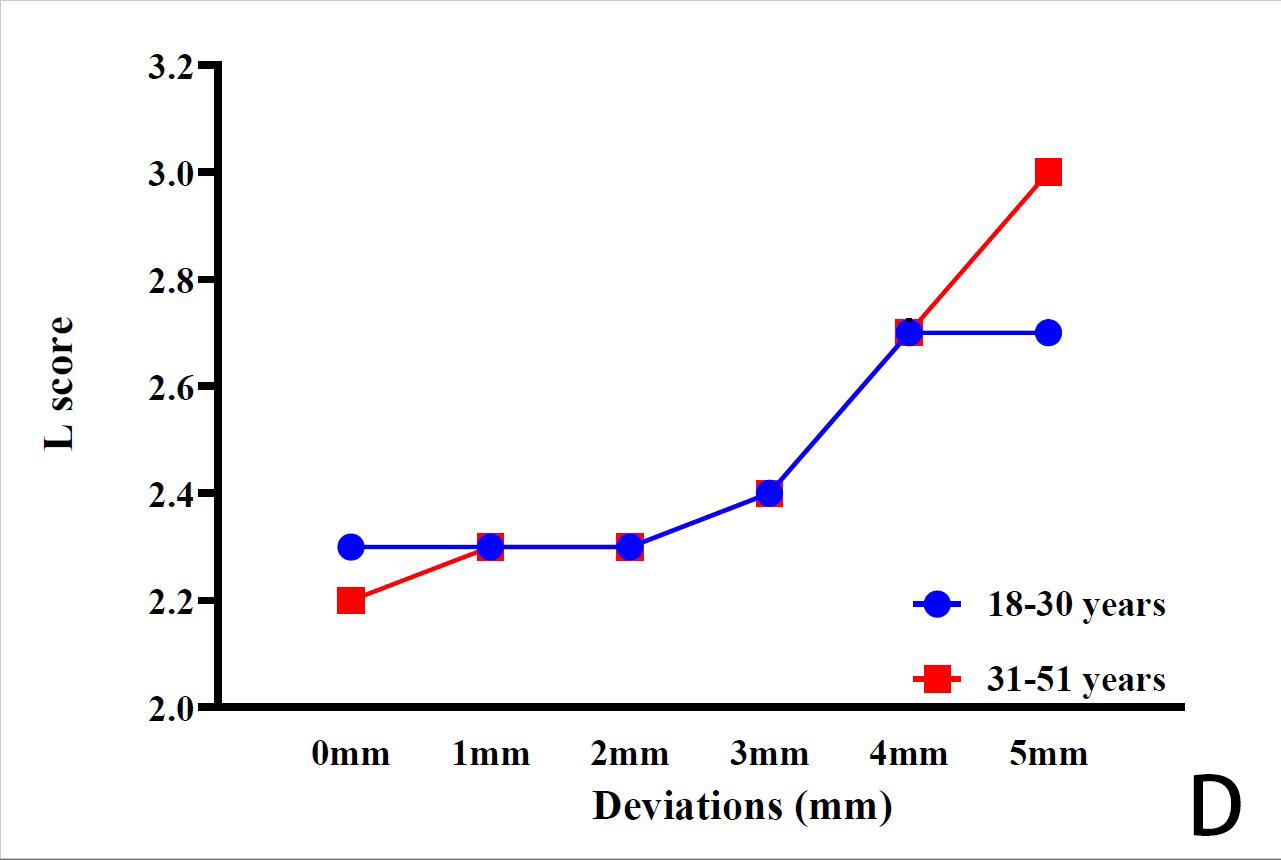 |
| 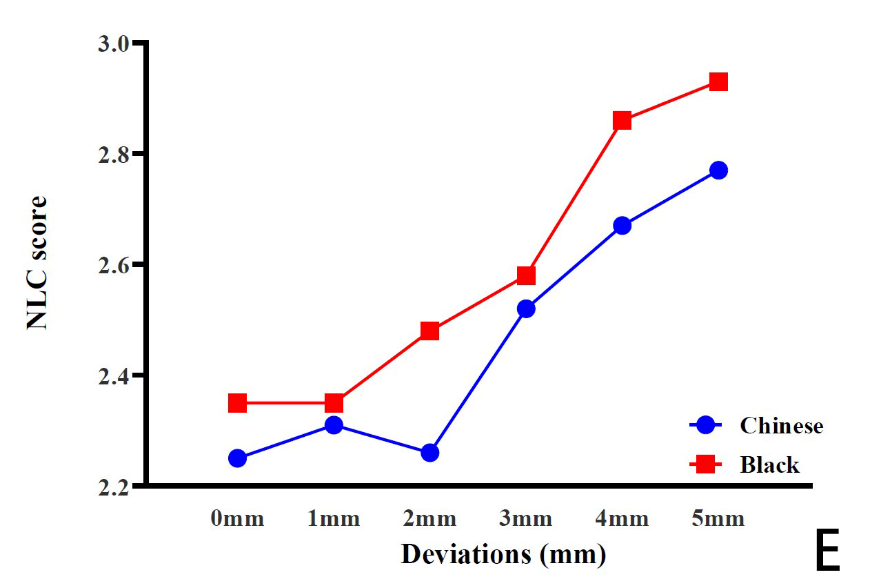 | 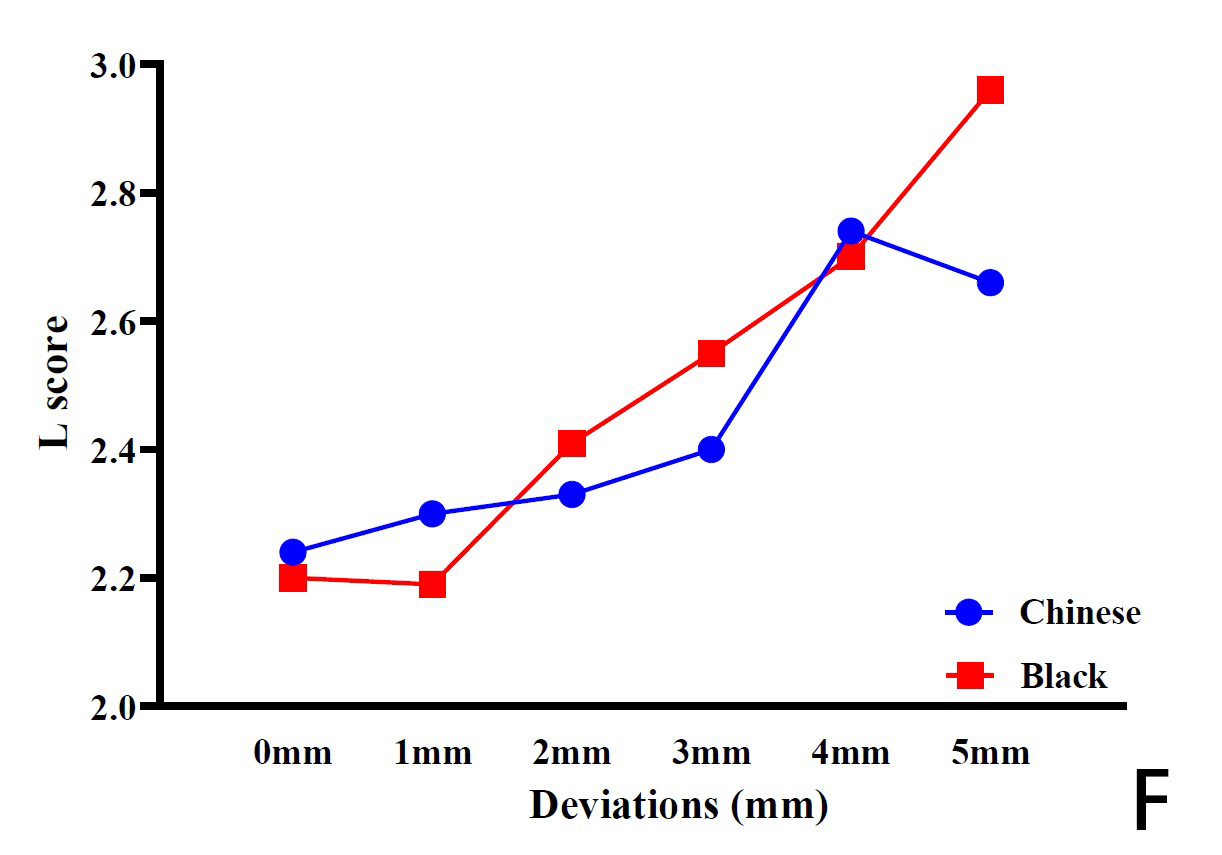 |

**Additional file 3.** Line graph showing the mean rating of attractiveness scores in NLC and L across categories of gender (**A**, **B**), age (**C**, **D**), and ethnicity (**E**, **F**) by overall raters (n=561). A higher score implies a less attractive smile
