# Supplementary material for: Effect of the ethnic, profession, gender, and social background on the perception of upper dental midline deviations in smile esthetics by Chinese and Black raters
Source: BMC Oral Health. 2023 Apr 14;23:214. doi: 10.1186/s12903-023-02893-4 (PMC10105468; doi:10.1186/s12903-023-02893-4)
Supplement: Supplementary file 4 — Additional file 4. [file 12903_2023_2893_MOESM4_ESM.docx]

| 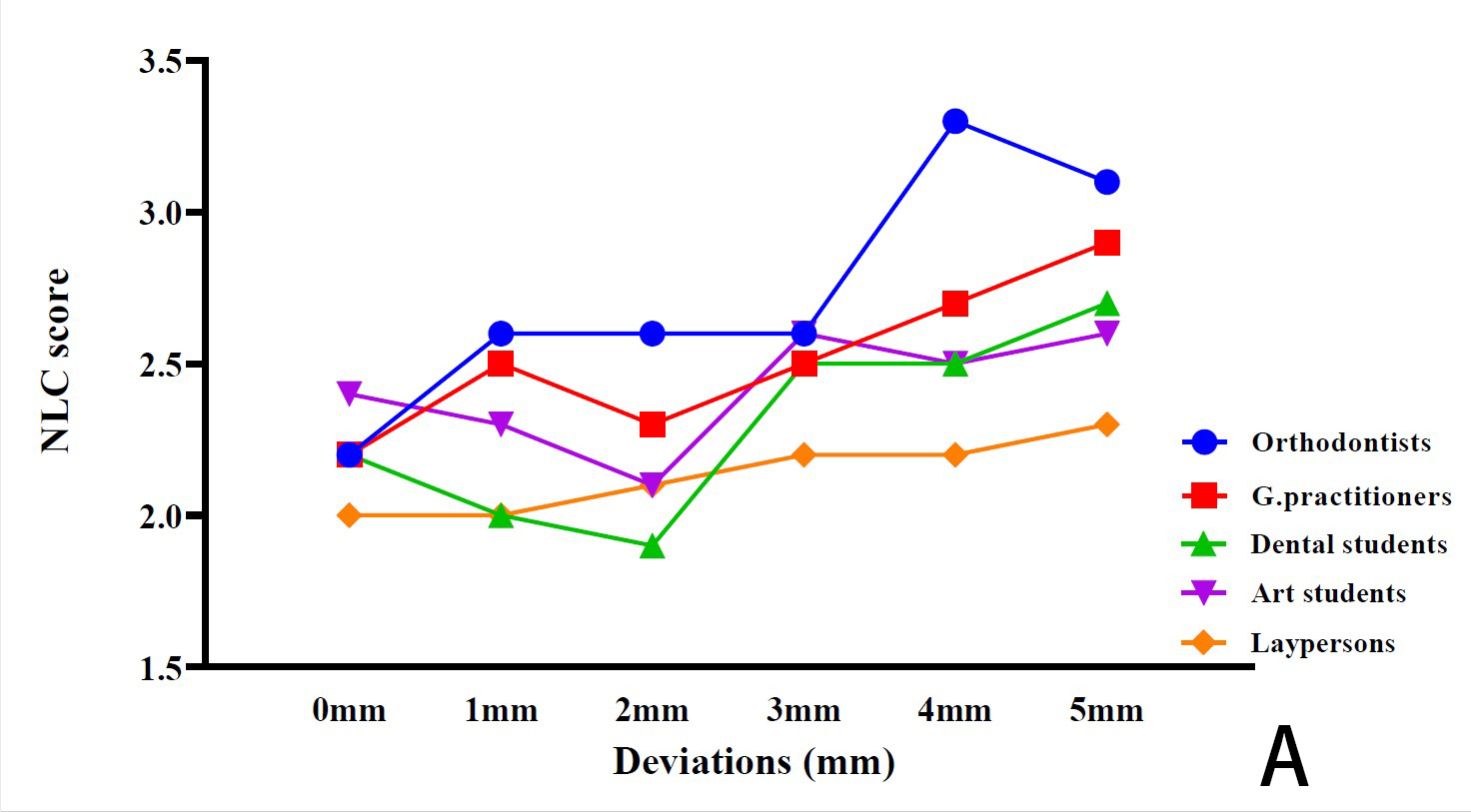 | 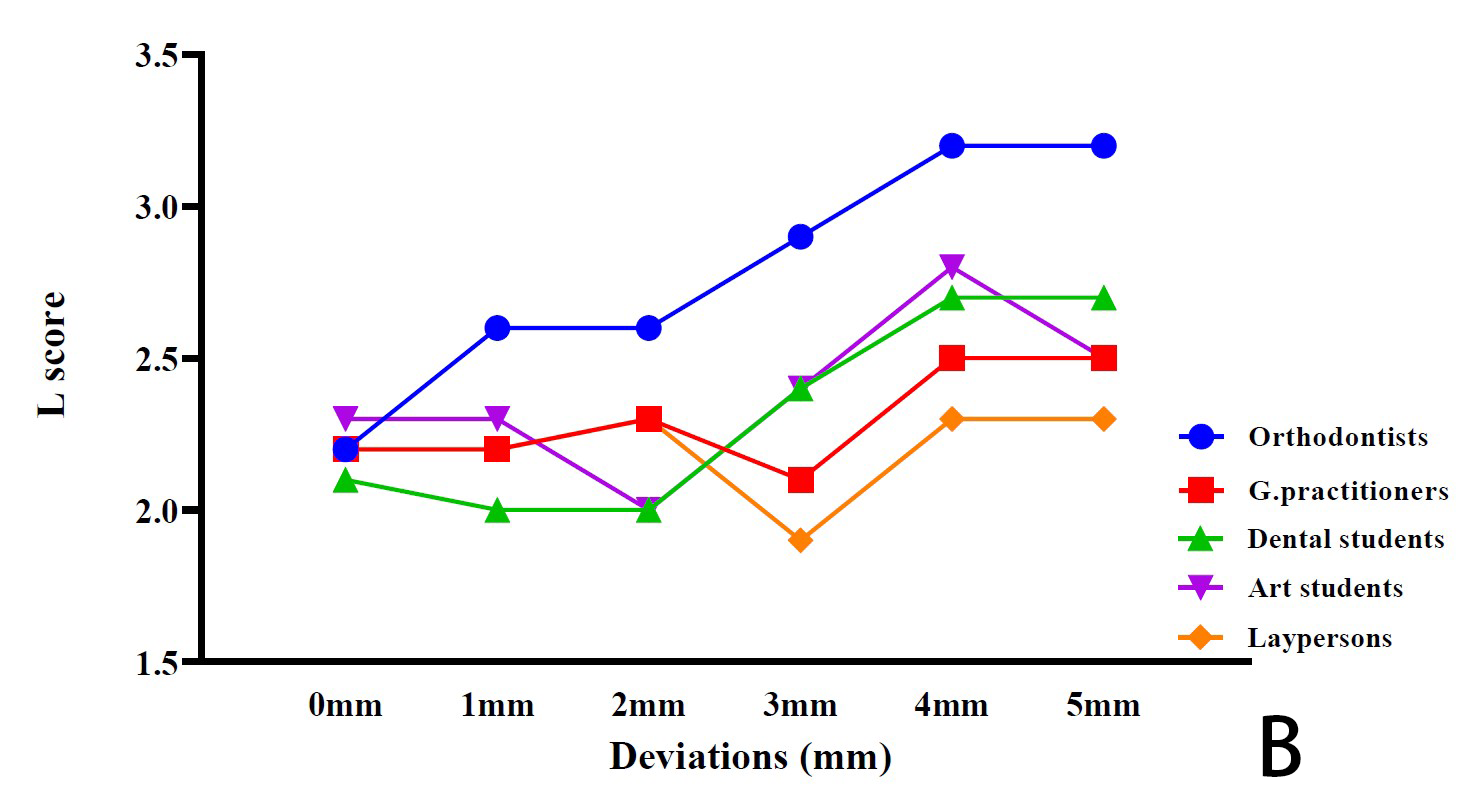 |
| --- | --- |
| 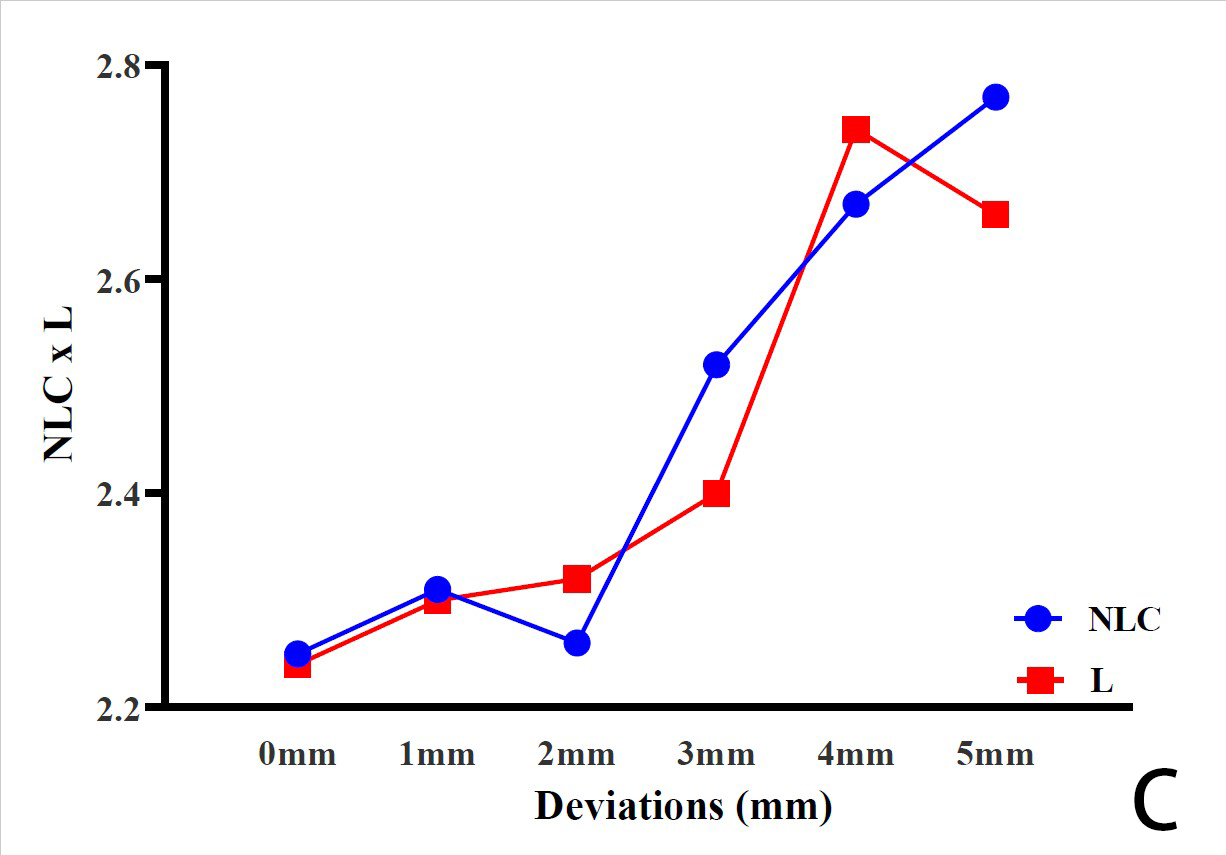 | 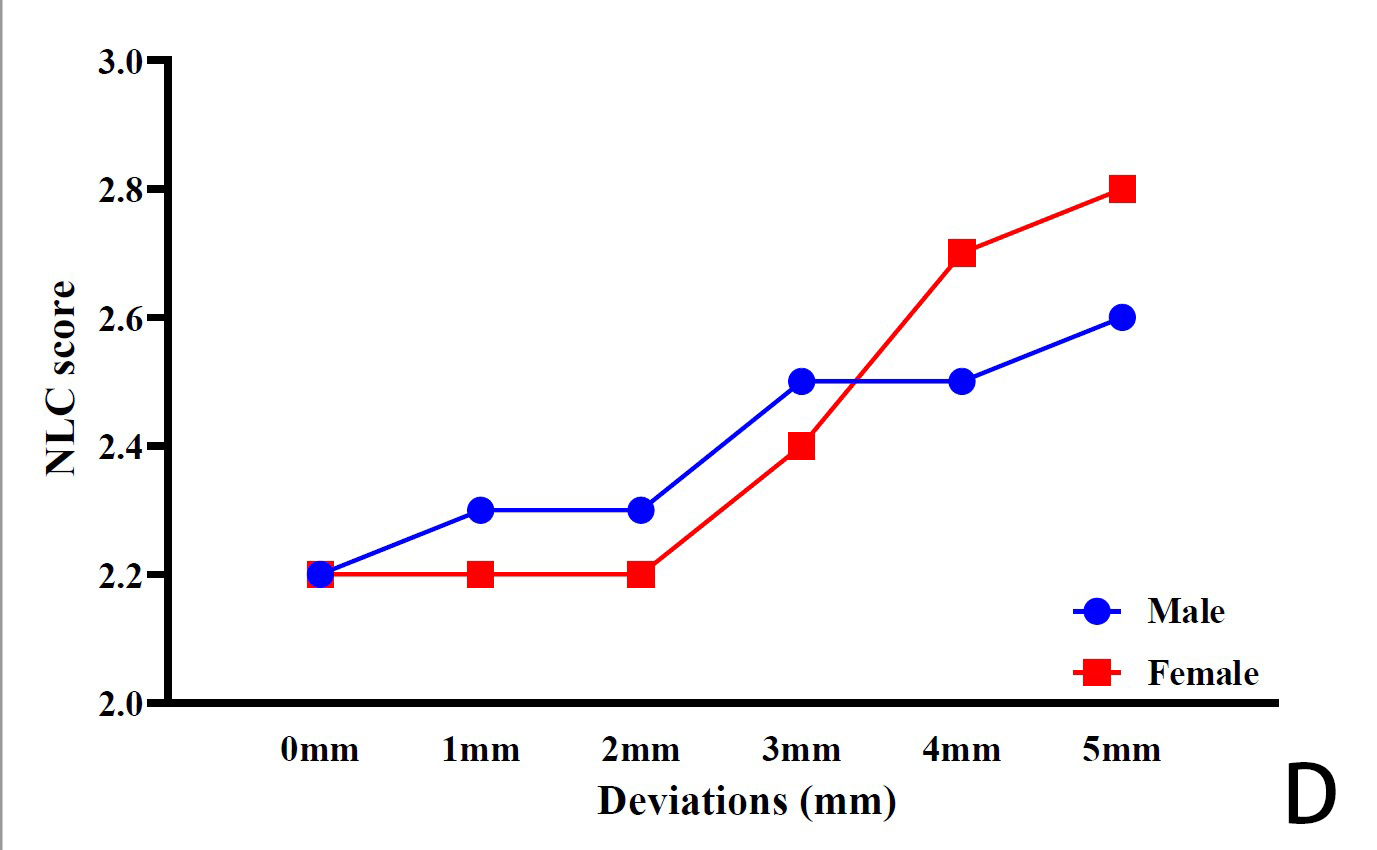 |
| 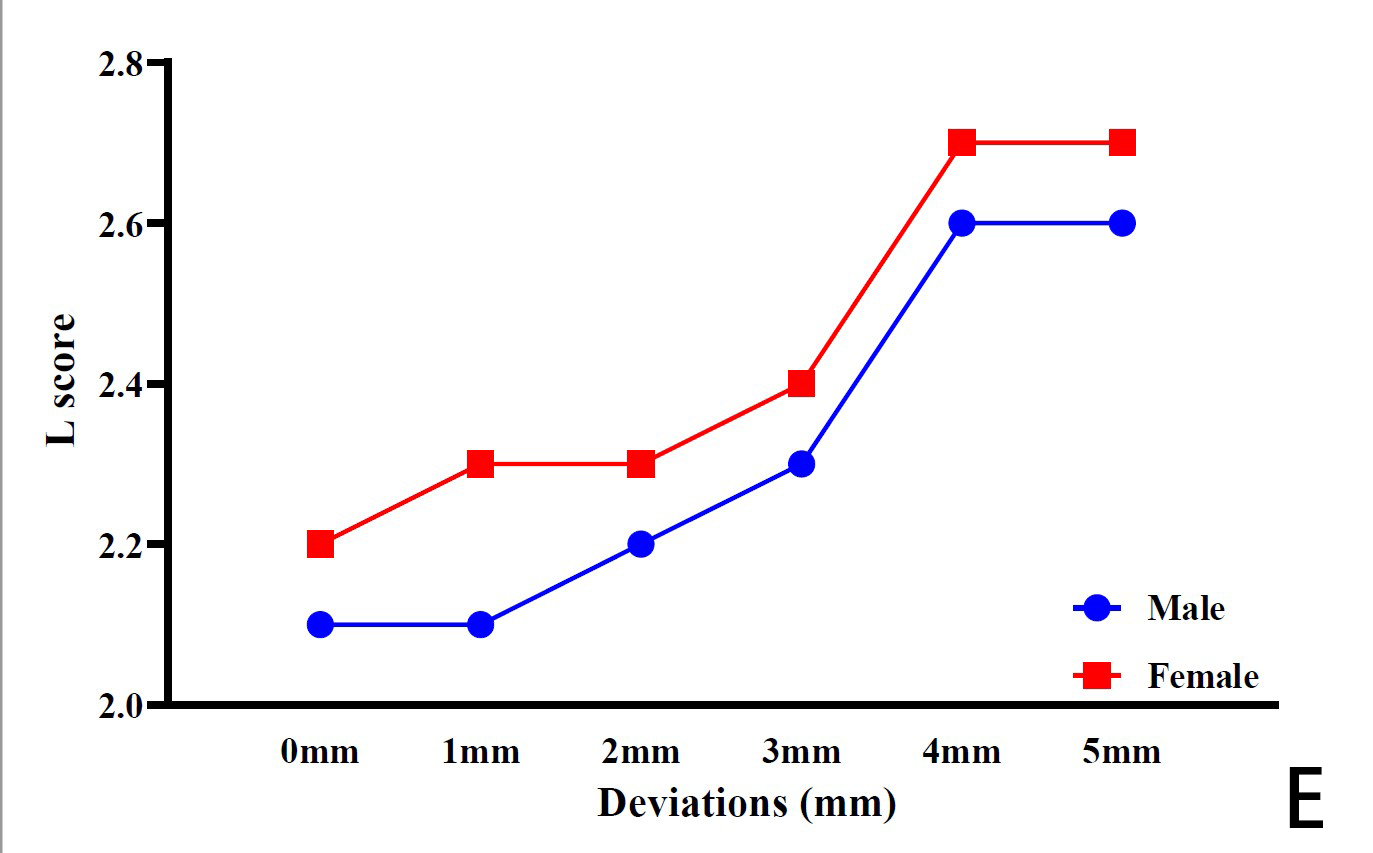 | 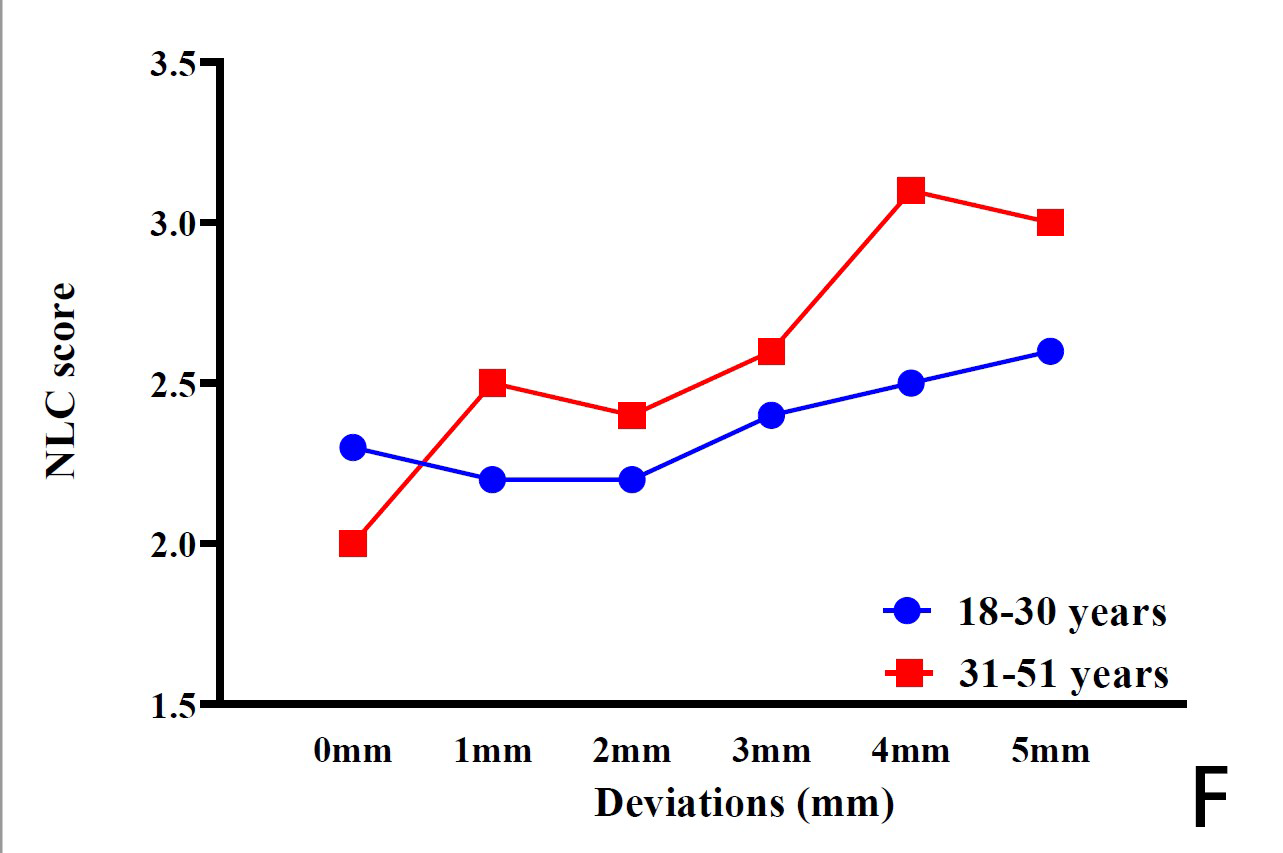 |
| 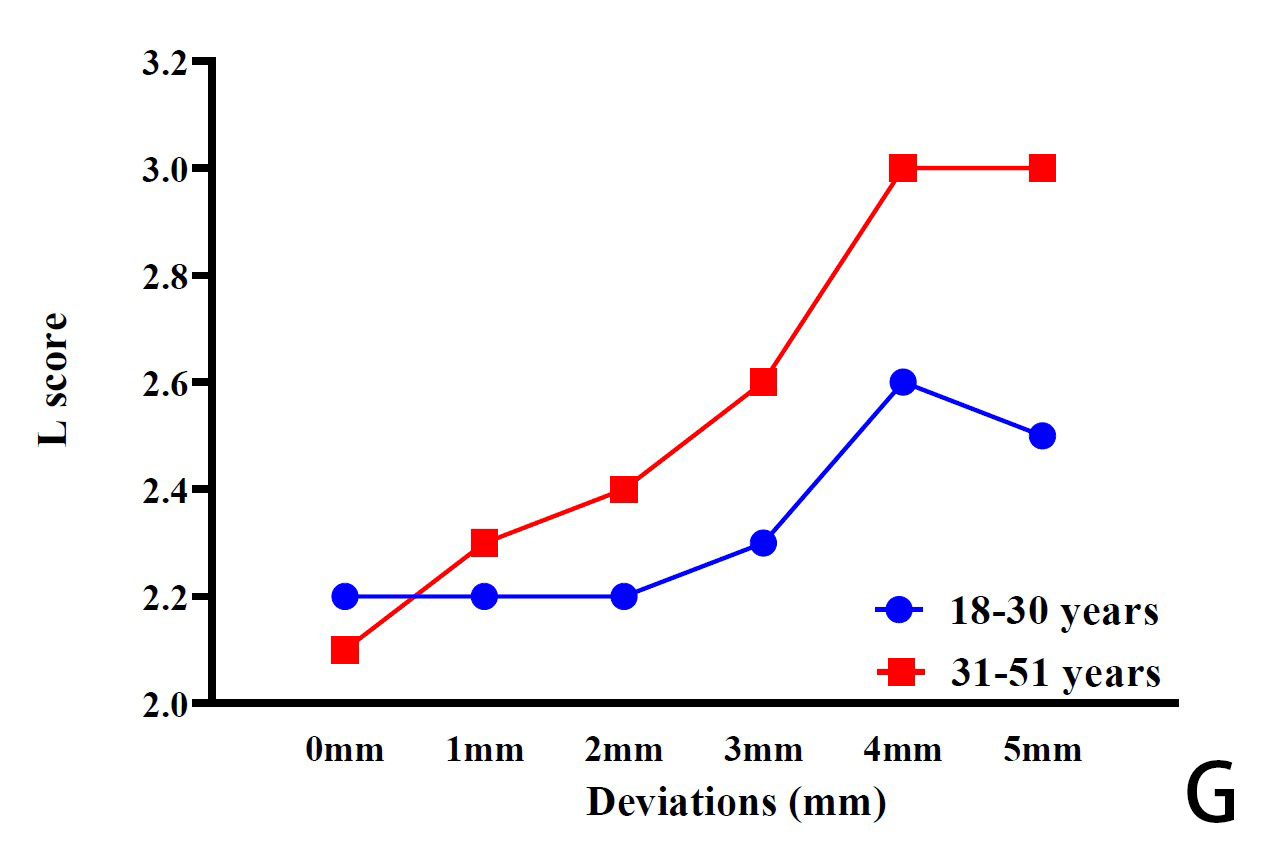 **E** |  |

**Additional file 4.** Line graph showing the mean rating of attractiveness scores in NLC and L across categories of the profession (A, B), smile-associated structure NLC x L (C), gender (D, E), and age (F, G) by Chinese raters (n=281). A higher score implies a less attractive smile
