# Supplementary material for: Effect of the ethnic, profession, gender, and social background on the perception of upper dental midline deviations in smile esthetics by Chinese and Black raters
Source: BMC Oral Health. 2023 Apr 14;23:214. doi: 10.1186/s12903-023-02893-4 (PMC10105468; doi:10.1186/s12903-023-02893-4)
Supplement: Supplementary file 6 — Additional file 6. [file 12903_2023_2893_MOESM6_ESM.docx]

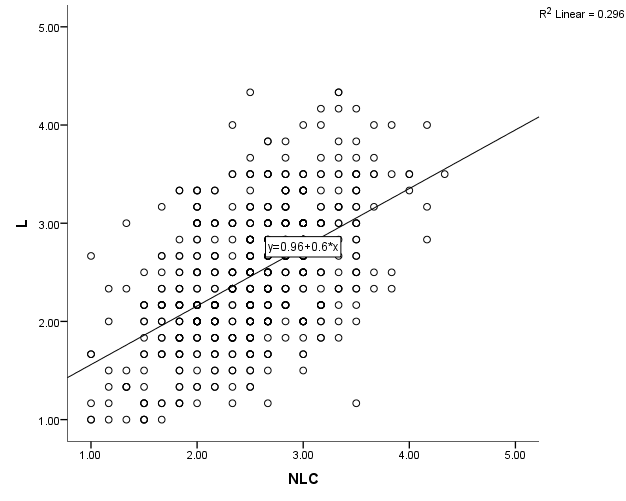


**Additional file 6.** A scatter plot shows the correlation between the deviations in (NLC and L) and the raters' mean values (n=561).
